# Complete Genome Sequence of the Intracellular Bacterial Symbiont TC1 in the Anaerobic Ciliate *Trimyema compressum*

**DOI:** 10.1128/genomeA.01032-16

**Published:** 2016-09-22

**Authors:** Naoya Shinzato, Hiroaki Aoyama, Seikoh Saitoh, Naruo Nikoh, Kazuma Nakano, Makiko Shimoji, Misuzu Shinzato, Kazuhito Satou, Kuniko Teruya, Takashi Hirano, Takanori Yamada, Masaru K. Nobu, Hideyuki Tamaki, Yumi Shirai, Sanghwa Park, Takashi Narihiro, Wen-Tso Liu, Yoichi Kamagata

**Affiliations:** a Tropical Biosphere Research Center, University of the Ryukyus, Nishihara, Okinawa, Japan; b Okinawa Institute of Advanced Sciences, Uruma, Okinawa, Japan; c Department of Liberal Arts, The Open University of Japan, Chiba, Chiba, Japan; d Bioproduction Research Institute, National Institute of Advanced Industrial Science and Technology (AIST), Tsukuba, Ibaraki, Japan; e Department of Civil and Environmental Engineering, University of Illinois at Urbana-Champaign, Urbana, Illinois, USA; f Center for Strategic Research Project, University of the Ryukyus, Nishihara, Okinawa, Japan

## Abstract

A free-living ciliate, *Trimyema compressum*, found in anoxic freshwater environments harbors methanogenic archaea and a bacterial symbiont named TC1 in its cytoplasm. Here, we report the complete genome sequence of the TC1 symbiont, consisting of a 1.59-Mb chromosome and a 35.8-kb plasmid, which was determined using the PacBio RSII sequencer.

## GENOME ANNOUNCEMENT

*Trimyema compressum* (Protozoa, Ciliophora) is an anaerobic free-living ciliate widely distributed in anoxic freshwater environments (1–3). *T. compressum* harbors a bacterial symbiont named TC1, which is a member of the *Firmicutes* and is distantly related to any known cultured strains, as well as methanogenic symbionts (4). The antibiotic treatment experiment suggested that the TC1 symbiont was crucial for vigorous growth of the ciliate, indicating that the relationship is mutualistic rather than parasitic (4). However, except taxonomic information, almost nothing is known about the TC1 symbiont to date. In the present study, we attempted to determine the genome sequence of the TC1 symbiont in order to understand its metabolism, physiological function, and evolution.

The cells of the TC1 symbiont were prepared from a total of 24 liters of ciliate cultures as follows. *T. compressum* was grown in synthetic medium supplemented with food bacteria (4). The ciliate cells were harvested at maximum cell density by centrifugation at 1,000 rpm for 5 min. After three times of washing with new medium, ciliate cells were ruptured by brief freezing at -80°C. The cell suspension was fractionated by passing through membrane filters with different pore sizes (in order of 60 µm, 11 µm, and 3 µm) to separate symbiont cells from the host’s cell debris. The symbiont cells in the flowthrough were collected by centrifugation. The genomic DNA of the symbiont was prepared by lysozyme and proteinase K treatments, according to the standard protocol (5).

Two micrograms of genomic DNA was subjected to 20-kb fragment library preparation and sequenced on PacBio RSII sequencer (Pacific Biosciences) using 16 single-molecule real-time (SMRT) cells and P6-C4 chemistry. *De novo* assembly using the HGAP/Quiver (6) workflow constructed two self-overlapping contigs. Each of the contigs was circularized with Minimus2 (7) and then was polished again with Quiver. In the final assembly, a 1,586,453-bp circular chromosome and 35,795 bp of plasmid sequences were obtained with 839× and 10,829× mean coverage, respectively. In the present study, any archaeal genome was not found in the contigs, although a number of short contigs derived from food bacteria were generated.

The G+C content of the chromosome was as low as 32.8%, which is a typical feature of the genome of intracellular symbionts. Preliminary gene annotation estimated the number of protein-cording genes in the chromosome to be 1,694. In addition, two rRNA gene operons and 36 tRNA genes were found in the chromosome. On the other hand, the plasmid also had a very low G+C content (29.7%) and encoded 40 proteins, including several phage-related genes, such as major capsid protein, tail proteins, and phage head maturation protease. A survey of insertion sequences (ISs) using ISfinder (8) predicted numerous IS-related open reading frames (ORFs) (35 complete and 147 partial ISs) in the chromosome. These results suggested that the TC1 symbiont is in the course of genome reduction process under the symbiotic lifestyle (9). Its complete genome sequence will give insight into the physiological role of the TC1 symbiont and evolutionary history of this tripartite symbiotic association.

### Accession number(s).

This whole-genome shotgun project has been deposited in DDBJ/ENA/GenBank under the accession numbers CP014606 (chromosome) and CP014607 (plasmid).

## References

[1] Wagener S, Pfennig N 1987 Monoxenic culture of the anaerobic ciliate *Trimyema compressum* lackey. Arch Microbiol 149:4–11. doi:10.1007/BF00423128.

[2] Goosen NK, Wagener S, Stumm CK 1990 A comparison of two strains of the anaerobic ciliate *Trimyema compressum*. Arch Microbiol 153:187–192. doi:10.1007/BF00247819.

[3] Yamada K, Kamagata Y, Nakamura K, Inamori Y, Nakamura I 1994 Selectivity of food bacteria for the growth of anaerobic ciliate *Trimyema compressum*. Arch Microbiol 161:229–233. doi:10.1007/BF00248697.

[4] Shinzato N, Watanabe I, Meng XY, Sekiguchi Y, Tamaki H, Matsui T, Kamagata Y 2007 Phylogenetic analysis and fluorescence *in situ* hybridization detection of archaeal and bacterial endosymbionts in the anaerobic ciliate *Trimyema compressum*. Microb Ecol 54:627–636. doi:10.1007/s00248-007-9218-1. 17468963

[5] Sambrook J, Fritsch EF, Maniatis T 1989 Molecular cloning: a laboratory manual, 2nd ed. Cold Spring Harbor Laboratory Press, Cold Spring Harbor, NY.

[6] Chin CS, Alexander DH, Marks P, Klammer AA, Drake J, Heiner C, Clum A, Copeland A, Huddleston J, Eichler EE, Turner SW, Korlach J 2013 Nonhybrid, finished microbial genome assemblies from long-read SMRT sequencing data. Nat Methods 10:563–569. doi:10.1038/nmeth.2474. 23644548

[7] Treangen TJ, Sommer DD, Angly FE, Koren S, Pop M 2011 Next generation sequence assembly with AMOS. Curr Protoc Bioinformatics Chapter 11:Unit 11.8. doi:10.1002/0471250953.bi1108s33. PMC3072823 21400694

[8] Siguier P, Perochon J, Lestrade L, Mahillon J, Chandler M 2006 ISfinder: the reference centre for bacterial insertion sequences. Nucleic Acids Res 34:D32–D36. doi:10.1093/nar/gkj014. 16381877 PMC1347377

[9] McCutcheon JP, Moran NA 2012 Extreme genome reduction in symbiotic bacteria. Nat Rev Microbiol 10:13–26. doi:10.1038/nrmicro2670. 22064560

